# Medical student wellness assessment beyond anxiety and depression: A scoping review

**DOI:** 10.1371/journal.pone.0276894

**Published:** 2022-10-27

**Authors:** Kay-Anne Haykal, Lara Pereira, Aidan Power, Karine Fournier

**Affiliations:** 1 Faculty of Medicine, University of Ottawa, Ottawa, ON, Canada; 2 Institute du Savoir Montfort, University of Ottawa, Ottawa, ON, Canada; 3 Health Sciences Library, University of Ottawa, Ottawa, ON, Canada; Manipal University College Malaysia (MUCM), MALAYSIA

## Abstract

**Background:**

A significant increase in distress and mental health illnesses has been identified in medical students during their training. As a result, medical schools have attempted to understand factors linked to well-being. Wellness questionnaires present a useful approach to identifying students with risk factors for mental health to provide appropriate resources for support and referrals. This study aims to identify validated questionnaires in the literature that measure medical student wellness.

**Methods:**

A scoping review methodology was selected and an exhaustive search of MEDLINE, Embase, CINAHL, APA PsycInfo, EPIC, and Education Source, was performed from 1999 to May 27, 2021. A compilation of validated wellness evaluation tools, surveys and questionnaires assessing wellness beyond depression and anxiety was reviewed. All validated methods of wellness assessment for medical students were included.

**Results:**

5,001 studies were identified once duplicate records were removed. After applying inclusion and exclusion criteria, 23 articles were included in a qualitative synthesis and explored in detail. The following six validated questionnaires measuring the wellness of medical school students are reported and discussed: the Medical Student Stress Profile (MSSP), the Medical Student Stress Questionnaire (MSSQ), the Medical Student Well-Being Index (MSWBI), the Perceived Medical School Stress (PMSS), the Perceived Stress Scale for Medical Students (PSSMS), and the Oldenburg Burnout Inventory—Medical Student Version (OLBI-MS). These validated questionnaires provide various aspects to the assessment of wellbeing.

**Conclusions:**

Wellbeing evaluations are reliable in identifying medical students who are at risk for mental health illnesses but must be chosen carefully based on contexts, academic environment and student population. A direct comparison between validated questionnaires for student wellbeing is not possible and individual medical schools must determine the appropriateness and validity of such tools based on population-specific characteristics and demands.

## Introduction

In the last decade, medical schools have increased their focus on wellness following significant psychological distress experienced by many students. Although there is no specific consensus on the definition of wellness, it is generally understood as an experience of satisfactory mental and physical health, a sense of purpose to life with satisfaction and meaning. The World Health Organization (WHO) defines health as ‘a state of complete physical, mental and social wellbeing and not merely the absence of disease or infirmity’ [1]. The term well-being refers to a positive rather than neutral state, framing health as a positive aspiration.

Approximately 50% of medical students demonstrate psychological helplessness, detachment and burnout [2]. At the start of their training, medical students have similar mental health to others of the same age and level of education. However, there is a significant decline in their mental health when starting medical school, and worsening as their progress in their training. As high levels of distress have been associated with burnout, depression, decreased empathy, unprofessional behavior, dropping out of medicine as well as suicidal risk, it is paramount to understand intrinsic and extrinsic factors linked to wellbeing. Personal factors such as personality traits, history of the disease, and types of coping mechanisms as well as institutional factors relating to competitiveness, excessive workloads, exposure to disease and absence of support have been associated with medical student decreased well-being [3].

Wellness evaluation is common among medical schools, especially utilizing tools assessing depression and anxiety. These allow programs to assess students’ mental health to identify struggling individuals and offer some type of support to help them overcome these difficulties. However, a gap in the literature exists as studies that systematically search for wellness evaluation tools to address wellness beyond depression and anxiety, have not been identified. It is extremely important to acknowledge that overall wellness goes beyond the lack of clinically diagnosed mental health conditions. Substance abuse, coping strategies, resiliency levels and general health must all be assessed to properly evaluate wellness as a whole.

Therefore, the objective of this study is to compile a comprehensive list of validated wellness evaluation tools and questionnaires assessing wellness beyond depression and anxiety via a scoping review. As the study was conducted, all previously validated methods of wellness assessment for medical students were included and assembled to provide institutions with a solid inventory of tools.

## Material and methods

This scoping review adhered to the Preferred Reporting Items for Systematic reviews and Meta-analyses–PRISMA checklist [4]. This research is exempt from the Research Ethics Board (REB).

### Identification of relevant studies

#### Eligibility criteria

Published articles using validated tools, questionnaires and surveys to evaluate medical student wellness around the world were included. The target population was undergraduate medical students. No language restrictions were set. Publications dates were set from 1999 to May 27^th^, 2021. The following scenarios were considered eligible, studies evaluating stress levels during the COVID-19 pandemic in medical students, effects of yoga on medical student wellness, and comparison of burnout levels between genders in medical students.

Studies were excluded if the wellness evaluation tool was not previously validated. Blogs, websites, and editorial and personal statements were not accepted. Studies evaluating wellness in students wanting to pursue a career in medicine but who are not registered in a medical program and postgraduate medical learners (fellows and residents) were not included.

#### Information sources & search tools

A search of the literature following a peer reviewed strategy [5] was conducted by an information specialist (K.F.) in MEDLINE(R) ALL (OvidSP), Embase (OvidSP), CINAHL (EBSCOHost), APA PsycInfo (OvidSP), ERIC (OvidSP), and Education Source (EBSCOHost) from 1999 to May 27^th^, 2021 using a combination of subject headings and keywords for the concept of “wellness”, “medical students” and “questionnaires” (S1 Appendix). In addition, Google Scholar was used as a grey literature search. All references were managed, and duplicate articles were removed automatically using Covidence [6] (Veritas Health Innovation, Melbourne, Australia) with a revision from two reviewers (L.P. and A.P.). Following the initial search of electronic search of databases, the authors discussed the breadth of the scope originally agreed upon requiring validated tools only for wellness assessments. As a result, following the guidance of the librarian, the authors agreed that additional exclusion criteria would be added. The additional criteria were that all articles which did not use a previously validated tool within the target population of medical students would be excluded.

### Study selection

A two-phase process was followed. In phase one, two reviewers (L.P. and A.P.) screened all titles and abstracts of the imported references independently. Those meeting the inclusion criteria were selected. In phase two, the same reviewers applied the inclusion criteria to the full form of all the articles having been included in phase one. Throughout phases one and two, conflicts between the reviewers were reconciled by discussing with the field expert and the first author (K-A.H.). The reference lists of the selected articles were manually screened to identify any relevant references that may have been missed during the search of electronic databases. Articles referenced by the field experts were also considered. The final selection and eligibility decisions were based on full-text articles.

### Charting the data

The “PICOS principle” was followed in the extraction of key features of the included articles [7]. Population: medical student; Intervention: Wellness evaluation using previously validated tools, questionnaires or surveys; Comparison: Not required/applicable; Outcome: Validated tool used to evaluate medical student wellness; Study design: No restriction to study design. The first author (K-A.H.) extracted all required data from included articles following a standardized form. The second author (L.P.) reviewed all retrieved information. Any conflicts were settled through a discussion between the reviewers.

## Results

### Study selection

**Fig 1:**
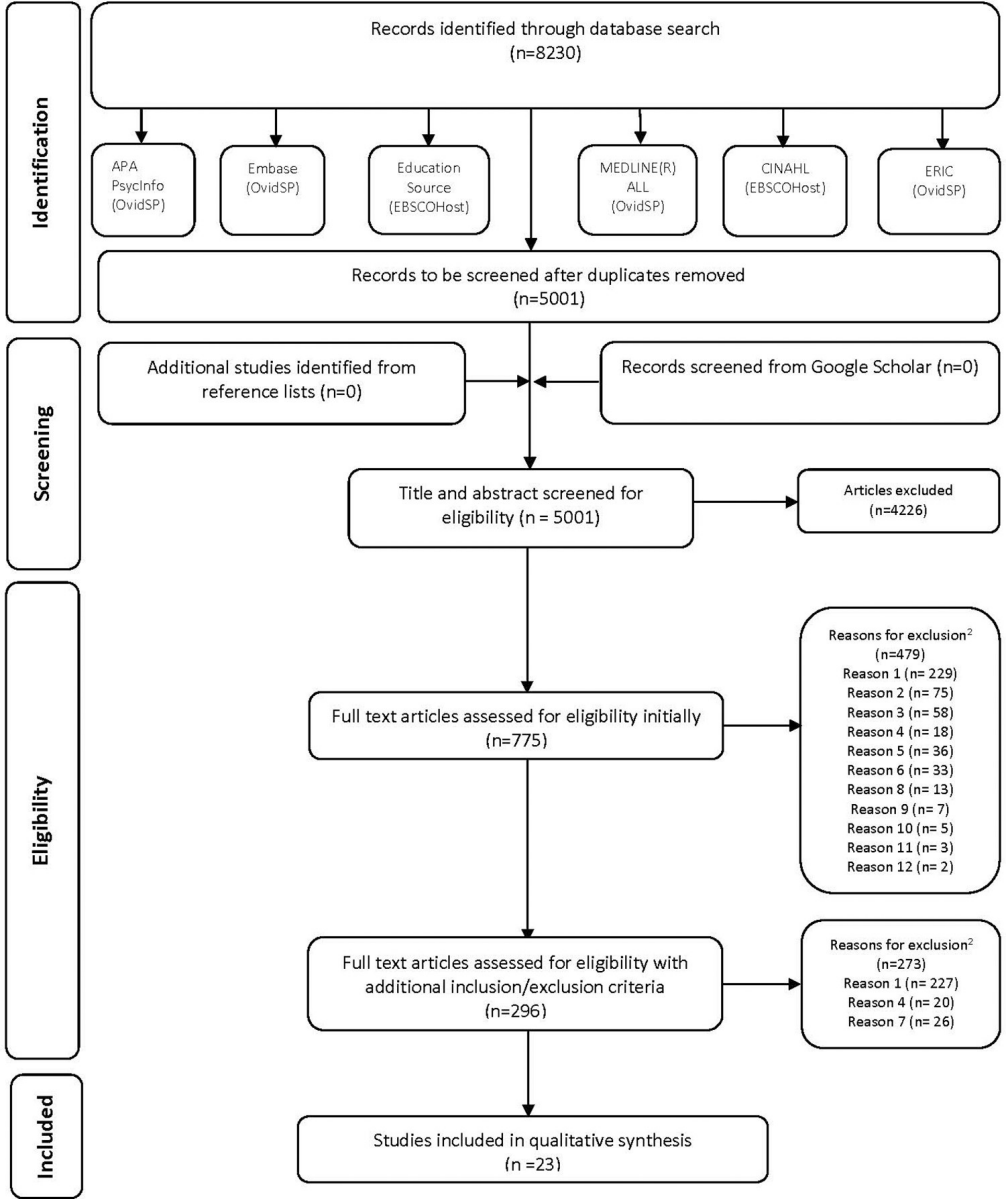
Flow diagram of literature search and selection criteria.

The electronic search of the databases identified 8,230 citations. 3,229 duplicate records were removed using Covidence which left 5,001 references for the screening phase. No additional studies were identified from the grey literature search. After eliminating 4226 articles, 775 articles entered phase two. The application of initial inclusion and exclusion criteria was applied and 479 articles were eliminated. A total of 296 articles were fully read, studied and assessed for eligibility. This number was deemed to be too large. Also, in many of these articles, the target population was students from different fields, and/or their measure of wellness had not been previously validated. Due to these findings, further inclusion and exclusion criteria were applied. The authors decided to include articles meeting an additional inclusion criterion, which was the use of previously validated tools as a measure of wellness within the medical school population specifically. Those additional criteria eliminated 273 articles. A total of 23 articles were included in the content qualitative synthesis and explored in detail. S2 Appendix shows texts not meeting the inclusion criteria and the reason for their exclusion. The flow diagram details the selection process, as per Fig 1.

Of the 23 articles, six unique, validated questionnaires measuring the wellness of medical school students were identified. Three of these questionnaires were used by multiple articles, while one article referred to each of the remaining three.

### Questionnaire findings

The Medical Student Stress Profile (MSSP) is a 100-item questionnaire developed in 2005 by O’Rourke et al. It measures students’ situational stress levels across five facets (time, supervision, course, social and patient-related stressors), coping mechanisms across four facets (emotion-focused, support-seeking, passivity, rationalization) and two measures of personal resources (negative affect and self-worth) [8]. It was not specified how stress was measured in their methods. One of the articles identified uses the MSSP [8].

The Medical Student Stress Questionnaire (MSSQ) is a 20 or 40-item questionnaire developed in 2010 by Yusoff et al [9]. It measures potential sources of stress on a 5-point Likert scale (“causing no stress at all = 0” to “causing severe stress = 4”) across six domains of related stressors: academic, intrapersonal and interpersonal, teaching and learning, social, drive and desire, and group activities [10]. The levels of stress were assessed with the MSSQ containing 40 items in six domains [9]:

Domain 1: academic-related stressors: tests/examinations, getting poor marks, falling behind in the reading schedule, unable to answer the teacher’s questions.

Domain 2: intrapersonal- and interpersonal-related stressors: need to do well, lack of time to review what has been learnt, having difficulty understanding the content.

Domain 3: teaching- and learning-related stressors: heavy workload, feeling of incompetence, participation in class presentation, participation in class discussion.

Domain 4: social-related stressors: frequent interruption of work by others, the unjustified grading process, facing illness or death of the patients.

Domain 5: drive- and desire-related stressors: not enough medical skill practice, verbal or physical abuse by teacher(s), talking to patients about personal problems.

Domain 6: group activities-related stressors: learning context–full of competition, quota system in examinations, verbal or physical abuse by other students.

The mean score of each of the six domains is calculated to determine the level of stress students are experiencing, classified as follows (0–1 = Mild, 1.01–2 = Moderate, 2.01–3 = High and 3.01–4 = Severe) [11]. Five of the articles identified using the 40-item version of the MSSQ [9, 12–15] while six used the shortened 20-item version [10, 11, 16–19].

The Medical Student Well-Being Index (MSWBI) is a 7-items questionnaire developed in 2010 by Dyrbye et al [20]. It measures potential sources of distress during the past month on a yes/no binary across five domains: burnout, stress, quality of life fatigue and depression [21]. To determine if a student is experiencing high levels of stress, one point is given for each “yes” answer and the total score from all seven responses is added up. Scores greater than or equal to four are indicative of a higher risk of severe distress, fatigue, burnout and potentially student dropout and suicide ideation [22]. Six of the articles identified to use the MSWBI [2, 21–25].

The Perceived Medical School Stress (PMSS) scale is a 13-item questionnaire developed in 1989 by Vitaliano et al [26]. It measures stress on a 5-point Likert scale ("strongly disagree = 0" to "strongly agree = 4") by asking questions about the medical school curriculum and environment, however, Voltmer et al (2012) altered their scale from 0–4 to 1–5 to compare results with a larger study. To determine if a student is stressed, all the responses are added together to calculate a total score, however, an exact threshold has not been determined [27]. The items explore themes such as isolation, perceived competition between classmates, subject matter mastery, time constraints and personal finances [27]. Three of the articles identified to use the PMSS [27–29].

The Perceived Stress Scale for Medical Students (PSSMS) is a 40-item questionnaire developed in 2010 by Mansoor et al to measure stressors faced by Pakistani medical students. The items are broken down into four subscales: 13 items for social stressors like interpersonal issues and self-worth/inferiority, 10 items for mistrust of peers or authority figures, 10 items for academic stressors like examinations and career prospects and seven items for burnout, including motivation and passive coping [30]. One of the articles identified uses the PSSMS [30].

The Oldenburg Burnout Inventory—Medical Student Version (OLBI-MS) is a 16-item questionnaire adapted from the general population Oldenburg Burnout Inventory, originally developed in German and translated to English in 2007 by Halbesleben et al. It has been used by the AAMC to measure the severity of medical student burnout along two dimensions of eight questions each: exhaustion and disengagement [31], on a 4-point Likert scale (“strongly disagree = 0” to “strongly agree = 3”). No exact threshold has been determined to definitively indicate burnout, but the higher the score is, the more likely it is that respondents are burnt out. Samuels et al. (2021) measured the score using a dichotomous variable that adds 12-points from the disengagement dimension and 13-points from the exhaustion dimension together on a scale from 0 to 24. One of the articles identified uses the OLBI-MS [31].

## Discussion

This study assessed seven validated questionnaires, which have been applied to an impressive total of over 37000 medical students, from 12 different countries. Well-being is now considered along multiple domains (emotional, physical, social, financial, etc.), providing a more holistic approach to one’s state of being. In an environment where academic achievement is not only well regarded and encouraged, but also required, psychological distress is associated with poor academic self-perception [22]. Mental well-being and other factors such as motivation or empathy are known to decrease as students progress in their medical education [29]. More importantly, one in five students had either taken or considered taking time off from medical school specifically for their wellbeing. Despite the important prevalence of psychological distress in medical students, efforts to address risk factors have largely been disregarded. Even today, medical student well-being remains poor, despite increased awareness, resources and attempts from faculties to increase mental health advocacy and support [24]. Additionally, it has been demonstrated that peer relationships can play a protective role against poor academic performance and distress [23]. Addressing mental health within medical institutions is a long-term investment to promote the growth of medical students into emotionally balanced physicians who contribute to the healthcare workforce.

### MSSQ assessed stress-related factors

The MSSQ was used in a versatile manner to assess, evaluate and correlate a multitude of stress-related factors in students. The most basic use of the MSSQ was to identify, recognize and quantify stressors [11, 15]. This goal was taken further in one article, where the information helped to initiate collaborative research between universities and facilitated the sharing of information by initiating strategies to reduce stress [14]. In some studies, the questionnaire aimed to correlate stress to health factors among students such as cardiovascular risk behaviours [13], ABO blood groups [18] and blood pressure [17]; where others simply identified stressors [16]. The MSSQ can evaluate the effects of both stressful events such as multiple mini-interviews and stress-reducing interventions on the stress and anxiety levels of students [10, 15]. Finally, the MSSQ-I was created from the MSSQ and implemented in a specific population [12].

The MSSQ was administered to over 3400 medical students in different academic years. One study compared first-year students at the beginning of their careers as a type of control to assess true levels of stress in students related to medical school in subsequent years [13]. Some studies evaluated students in only one specific year [15, 16, 19], and others included students from various academic years [9, 11–13].

It has been demonstrated that the division of the population among each participant’s respective year allows for stratification of results and therefore comparison of variables among populations from different stages of their education. Some results specified differences based on the academic year of study. This can be explained by different coping mechanisms, previous experiences in students with more advanced education versus newly admitted students, and different demands of each academic year [9, 13]. The division of the population and analysis of stress stratification can be extremely beneficial for universities to promote stress-relieving resources that are tailored to the specific requirements of each academic year. For example, Hanakova et al (2015) found that first-year students are more at risk of experiencing stress due to their lack of previous experience with medical school. However, Yousoff et al (2010) had conflicting results and portrayed a higher level of stress among second and fourth-year students. Although the year of study was the main significant factor influencing stress, more research must be done to clarify this discrepancy in findings. Moreover, students from different nationalities experienced the same stress levels [13] but the cultural difference in the stress was related to the medical education system, as the above results originate from studies conducted in Malaysia and the Czech Republic.

Domain 1 of the MSSQ which includes the academic-related factors, was found to be among the most important stressors in a few studies [10, 11, 13, 16] and the utmost important factor causing stress, which is consistent with those presented in other studies from different countries [9, 17].

The MSSQ has been used in its entirety, a 40-items questionnaire, but also a shortened and validated version of 20 items, the MSSQ-20. Although none of the included articles described a difference between the two versions, it is possible to speculate that some institutions might prefer to use the shortened version for efficiency and response rate purposes, while others could benefit from a more exhaustive questionnaire.

The utilization of the MSSQ to assess stress levels is the main strength of the methodology of the two included studies [17, 18]. As this questionnaire was validated, it recognized the specific source of stress for medical students. More importantly, this survey has been employed in multiple populations, among those are medical students in India, Nepal, Malaysia, Italy and the Czech Republic.

The MSSQ-I has 37 items instead of 40; one item was discarded during the cultural validation and the last two items were deleted for unspecified purposes [12]. This exclusion can be explained by the differences in the educational curriculum for their Italian population versus the original MSSQ population in Malaysia. Dagani et al (2020) describe class presentations and discussions as being unusual within the Italian medical education system. Therefore, some aspects of the MSSQ related to stress during public speaking, presentations and discussions may not be relevant. The ability to validate a questionnaire within a population by making that tool more personable and culturally relevant may allow more populations to be assessed while maintaining internal validity.

Multiple studies mention the lack of generalizability as one of the weaknesses of their methodology [10, 16, 19]. As the population on which MSSQ is assessed is limited to medical students, the study context must be considered. Medical education is heterogeneous around the world and results may be different in variable educational settings [10]. Furthermore, the MSSQ is a ‘snap-shot observation’, as described by Yousoff et al (2020) and consequently, results may fluctuate over time [19]. In addition, medical school is a long process consisting of many distinct temporally sequential steps, each having its obstacles and stressors. Battula et al (2021) further state that despite assuring anonymity and confidentiality of the survey results, participants could under-report many of the stressors for a multitude of reasons.

### MSWBI focused on wellbeing

The MSWBI has been described as a powerful risk stratification tool [21] and a robust measure of medical student wellbeing [2]. Although the instrument is validated as a screening tool, it is not a diagnostic measure for the domains it assesses. One study suggests that it is a satisfactory tool to screen and monitor trends over time [23]. Another confirms that the MSWBI had shown acceptable levels of psychometric properties [25].

Different types of objectives were set around the use of MSWBI among medical students. Originally, it was created to identify domains of fatigue, mental quality of life and stress, and other mental health aspects. In two of the included articles, the main objective was to evaluate the performance of the questionnaire and its validity [15, 21]. Others studied the effect variables such as time [23] and interventions like peer support [22] on mental health. Interestingly, one study evaluated the correlation between well-being before Step 1, which is a significant exam that American second-year students must complete, to the subsequent performance on the test [2].

In comparison to other surveys not included in this article but pertinent to assess mental health such as the DASS-21 (depression and anxiety severity scale– 21 items), one author describes the MSWBI as an equally established instrument to assess psychological distress, anxiety, depression and stress [25]. Also, scores in the DASS-21 and the MSWBI seem to be directly correlated, as high scores in one are associated with high scores in [25]. One weakness surrounding the use of MSWBI not specific to the tool, as reported by Rajapuram et al (2020), is the inability to track individual responses in a large population.

### PMSS for behavior

Articles using the PMSS for behavior questionnaire [27, 29] sought to examine medical students’ behavior and experience pattern development over time [29], to establish levels of psychological distress [28], and to compare distress levels between groups of students having been exposed to an intervention versus a control group [27]. In comparison to other questionnaires, the PMSS also assesses potential stressors specific to medical school [28]. However, unlike other questionnaires, the PMSS includes specific factors such as lack of time for social and recreational activities, worries about finances and accommodations within the assessment of wellness [27]. Similarly, the PMSS scores have been found to anticipate mental health problems that require treatment specifically and represent a vulnerability measure by significantly predicting suicidal behaviors in medical students [28]. This is contrasted with other previously mentioned surveys such as the MSSQ which assesses stressors [11, 15] and the MSWBI which identifies psychological distress [21].

### PSSMS

One article in our selection [30] used the PSSMS questionnaire. The goal was to study how stressors perceived by medical students predict suicidal behavior in them. Similarly, to the MSWBI and PMSS, which measured suicidal behaviors or ideation, the PSSMS addressed four subscales of Social Stressors, Mistrust, Academic Stressors, and Burnout. The total score of the Perceived Stress Scale by medical students significantly predicted about suicidal behaviors in medical students. This tool is useful to faculties who intend to study, address and minimize suicidal behaviors among their medical students, especially in a country like Canada where up to 6.4% of medical students engage in self-reported suicide attempts [32].

### MSSP for stress

Only one article [8] employed the MSSP to demonstrate its psychometric quality as a specific device for auditing medical student stress. The aim was to establish the reliability, construct and criterion validity of this instrument; and to explore the relationships between stress, coping, personality, motivation and emotional intelligence in medical students. Interestingly, the MSSP is a tailor-made instrument tool that targets situation-specific stressors. As previously described by Yusoff (2020), the study context must be considered when assessing medical students’ mental health.

### OLBI-MS

One article presented the OLBI-MS [31], which was adapted from the Oldenburg Inventory and used by the AAMC, medical educators, and researchers to assess the severity of student burnout. With this tool, it was demonstrated that LGT (lesbian, gay, transgender) students who experienced mistreatment specific to their sexual orientation had more than eight times a higher probability of burnout in comparison to other students. As a result, the OLBI-MS provides an association between burnout and perceived mistreatment specific to gender across different student populations, associating more importantly that the context has to be considered in the measurement of wellness [10].

Overall, these validated questionnaires provided a broad overview of the various aspects of well-being. Tools must be chosen with attention to specific contexts, academic environments and student populations as described above, the strength of our study is the breadth of the search, dating back many decades and leading to a systematic coverage of all the published material. This ensures that no articles are missed and that all available and eligible surveys are assessed. Furthermore, surveys were only deemed eligible if they were previously validated onto the target population. This enhances the quality of our findings to allow faculties to search and utilize the appropriate tools within their context. The above review of validated questionnaires presents an efficient, reliable and useful source for identifying the most applicable assessment for medical students.

However, a few limitations should be discussed. It is impossible to fully assess adequately and equally report on the validated surveys included. The main goal of this article was the gathering and description of published validated surveys, which are available to assess the well-being of medical students. However, the authors are unable to provide detailed and consistent descriptions regarding each survey because these descriptions are dependent on the original initial presentation. Additionally, it is difficult to recommend one questionnaire as the most appropriate tool is specific to time, context and specific. Finally, the significant inconsistency in the quantity and quality of the descriptions of each questionnaire does not allow direct comparison between articles.

## Conclusion

In conclusion, medical students face significant stressors during their education and transitions from an undergraduate degree to residency. In anticipation of many barriers and the repercussions on their mental health, medical students can provide a structured wellness evaluation for identification, monitoring or follow-up of these learners. This systematic search for validated wellness questionnaires was able to identify the six above tools and discuss their strength and limitations. Medical faculties can determine the appropriateness and validity of each questionnaire within their specific context to define and provide appropriate resources and supports identified.

## Supporting information

S1 Checklist PRISMA. (DOCX)

S1 Appendix Search strategy. (DOCX)

S2 Appendix Articles excluded and the reasons for exclusion. (DOCX)
