# Supplementary material for: Medical student wellness assessment beyond anxiety and depression: A scoping review
Source: PLoS One. 2022 Oct 27;17(10):e0276894. doi: 10.1371/journal.pone.0276894 (PMC9612562; doi:10.1371/journal.pone.0276894)
Supplement: S1 Appendix — (DOCX) [file pone.0276894.s002.docx]

Search strategy

Search performed May 20, 2021

MEDLINE(R) ALL (OvidSP, 1946 to May 19, 2021)

1. Students, Medical/ or Schools, Medical/

2. education, medical/ or education, medical, undergraduate/

3. (medical education or medical school* or (medic* adj1 facult*)).ti,ab,kf.

4. UGME.ti,ab,kf.

5. ((medical or medicine) adj1 (student* or trainee* or undergrad*)).ti,ab,kf.

6. or/1-5

7. Mental Health/

8. risk/ or risk assessment/ or risk factors/

9. 7 and 8

10. Psychological Distress/ or exp Burnout, Psychological/

11. (mental health adj3 (risk* or distress* or concern* or problem* or issue* or status* or condition* or state*)).ti,ab,kf.

12. (psychological distress* or burnout*).ti,ab,kf.

13. (wellness* or well being* or wellbeing*).ti,ab,kf.

14. or/9-13

15. "Surveys and Questionnaires"/

16. psychiatric status rating scales/ or brief psychiatric rating scale/ or Psychological Tests/ or Psychometrics/

17. (Questionnaire* or survey* or form? or tool* or instrument* or test* or scale* or inventory or inventories or checklist* or index or indexes or assessment* or evaluat* or screening or measur*).ti,ab,kf.

18. (MSWBI or MS-WBI).ti,ab,kf.

19. or/15-18

20. 6 and 14 and 19

21. limit 20 to yr="1999-Current"

Results: **2252** references retrieved

Strategy developed by Karine Fournier.

In collaboration with Dr. Kay-Anne Haykal, Lara Pereira, Aidan Power, University of Ottawa, ON, Canada

May, 2021.
