# Supplementary material for: Medical student wellness assessment beyond anxiety and depression: A scoping review
Source: PLoS One. 2022 Oct 27;17(10):e0276894. doi: 10.1371/journal.pone.0276894 (PMC9612562; doi:10.1371/journal.pone.0276894)
Supplement: S2 Appendix — (DOCX) [file pone.0276894.s003.docx]

**Appendix 2 -** Articles excluded and the reasons for exclusion.

| Reason | Reasons for exclusion* | Number of articles |
| --- | --- | --- |
| 1 | No validated measure of wellness for medical students | 456 |
| 2 | No full text available | 75 |
| 3 | Wrong objective | 58 |
| 4 | Wrong population | 38 |
| 5 | Duplicate | 36 |
| 6 | Full text in foreign language | 33 |
| 7 | No validated measure of wellness | 26 |
| 8 | Unvalidated shortened/modified version of measure of wellness | 13 |
| 9 | Reply to editor or comment or revision | 7 |
| 10 | Abstract/poster presentation | 5 |
| 11 | Title/abstract in different language | 3 |
| 12 | Review | 2 |
